# Calcium Cyanamide as an Alternative Nitrogen Fertilizer: A Comprehensive Review of Its Agronomic and Environmental Impacts

**DOI:** 10.3390/plants15050673

**Published:** 2026-02-24

**Authors:** Mzwakhile Petros Zakhe Simelane, Puffy Soundy, Martin Makgose Maboko

**Affiliations:** 1 Department of Crop Sciences, Tshwane University of Technology, Private Bag X680, Pretoria 0001, South Africa; mzwakhezakhe5@gmail.com (M.P.Z.S.); soundyp@tut.ac.za (P.S.); 2 Department of Agriculture and Animal Health, University of South Africa, Roodepoort 1709, South Africa

**Keywords:** leaching, nitrogen use efficiency, slow-release fertilizer, soil-borne pathogens

## Abstract

Calcium cyanamide (CaCN_2_), commercially known as Perlka^®^, is re-emerging as a multifunctional nitrogen (N) fertilizer with significant agronomic and environmental advantages. Composed of 19.8% nitrogen and 50% calcium oxide (CaO), CaCN_2_ not only supplies slow-release nitrogen but also acts as a liming agent, improving soil pH and structure. Its transformation pathway: cyanamide → urea → ammonium → nitrate—ensures a gradual nitrogen release that aligns with crop demand, enhances nitrogen use efficiency, and minimizes nitrate leaching and nitrous oxide emissions. Additionally, the presence of dicyandiamide, a known nitrification inhibitor, further stabilizes nitrogen in the soil. Field studies across diverse cropping systems, including curly endive and short-day onions, have demonstrated that CaCN_2_ improves yield, crop quality, and soil health. In onions, preplant application of 80 kg ha^−1^ N from CaCN_2_ increased bulb yield by up to 18%, enhanced phytochemical content (e.g., phenolics and flavonoids), and reduced nitrate leaching by over 40% compared to urea and limestone ammonium nitrate (LAN). In curly endive, CaCN_2_ significantly improved ascorbic acid, total soluble solids, and phenolic content, particularly in fall-grown crops, while reducing nitrate accumulation and improving physiological and recovery efficiency of applied nitrogen. Beyond its role as a nutrient supplier, CaCN_2_ exhibits biocidal properties that suppress soil-borne pathogens such as *Sclerotinia* and *Plasmodiophora brassicae*, reduce weed pressure, and stimulate beneficial microbial activity. Its high calcium content also addresses physiological disorders linked to calcium deficiency, such as tip-burn and blossom-end rot. However, proper application timing and dosage are critical to avoid phytotoxicity, especially in sensitive crops. This review synthesizes current knowledge on CaCN_2_’s chemical behavior, agronomic performance, and environmental implications, and identifies research gaps to guide its optimized use in climate-smart and resource-efficient agriculture.

## 1. Introduction

Nitrogen (N) fertilization remains a cornerstone of modern agriculture, profoundly influencing both the yield and nutritional quality of vegetable crops [1]. As a fundamental component of amino acids, proteins, nucleic acids, chlorophyll, and various secondary metabolites, nitrogen is indispensable for plant metabolism, growth, and development [2]. Its strategic application underpins global food security by supporting intensive crop production systems. However, the widespread and often excessive use of conventional nitrogen fertilizers has led to a cascade of environmental challenges, including nitrate leaching, greenhouse gas emissions, and eutrophication of aquatic ecosystems [2,3,4]. These issues are particularly pronounced in developing regions, where limited access to precision agriculture tools and erratic climatic conditions exacerbate nitrogen inefficiencies and ecological degradation.

In response to these concerns, there is an urgent need for sustainable fertilization strategies that reconcile productivity with environmental stewardship. Conventional nitrogen sources such as urea and ammonium nitrate, while effective in promoting rapid vegetative growth, are highly soluble and quickly converted into nitrate, a mobile form that is prone to leaching, volatilization, and denitrification [5]. Alarmingly, it is estimated that only 30–50% of applied nitrogen is absorbed by crops, with the remainder lost to the environment, resulting in both economic inefficiencies and ecological harm [6].

Among the emerging alternatives, calcium cyanamide (CaCN_2_), commercially known as Perlka^®^ (produced by AlzChem, Trostberg, Germany), is gaining renewed attention as a multifunctional nitrogen fertilizer. First synthesized in the late 19th century, CaCN_2_ contains 19.8% nitrogen and offers a slow-release profile that aligns nutrient availability with crop demand [7]. Beyond its role as a nitrogen source, CaCN_2_ functions as a soil conditioner and a biocontrol agent. Historically used in Europe under the trade name Azotniak, it was effective in weed suppression and soil disinfection, although its powdered form posed handling challenges that led to its decline in use [7,8,9]. Modern formulations have addressed these limitations, reintroducing CaCN_2_ in granulated form suitable for precision agriculture in a wide range of vegetable crops [10].

Agronomically, CaCN_2_ offers several advantages over conventional fertilizers. It supplies calcium, which acts as a liming agent to neutralize soil acidity and improve soil structure [11]. Its slow hydrolysis in moist soil releases cyanamide, which is gradually converted to urea, ammonium, and nitrate, thereby reducing nitrogen losses and enhancing nitrogen use efficiency [12]. Moreover, CaCN_2_ acts as a natural nitrification inhibitor, delaying the microbial conversion of ammonium to nitrate and thus minimizing leaching and gaseous losses [13,14].

Beyond nutrient management, CaCN_2_ contributes to integrated pest and disease control [15]. It has been shown to suppress weed seed germination, reduce populations of soil-borne pathogens such as *Sclerotinia sclerotiorum* and *Fusarium oxysporum*, and mitigate nematode infestations [16,17]. These properties are particularly valuable in intensive vegetable systems with limited crop rotation, where pest and disease pressures are high.

Recent field studies have consistently reinforced the agronomic and environmental advantages of calcium cyanamide (CaCN_2_) across a variety of crops and growing conditions. In curly endive (*Cichorium endivia* L.), Sabatino et al. [18] demonstrated that pre-transplant application of CaCN_2_ at 100 kg ha^−1^ N significantly enhanced functional quality traits, including ascorbic acid, total phenolic content, and total soluble solids, compared to conventional ammonium nitrate fertilization. Notably, CaCN_2_-treated plants exhibited lower nitrate accumulation and higher physiological nitrogen use efficiency (PE_N), particularly in fall-grown crops, suggesting improved nitrogen synchronization and reduced environmental losses. Similar improvements in crop quality and nitrogen efficiency have been reported in lettuce [19] and strawberry [20], further validating CaCN_2_’s multifunctional role in horticultural systems.

Complementary findings in short-day onion production further underscore CaCN_2_’s efficacy. Simelane et al. [21] reported that preplant application of 80 kg ha^−1^ N from CaCN_2_ significantly increased bulb yield, reduced nitrate leaching, and minimized premature bolting compared to urea and LAN application. When combined with topdressings of urea or LAN, CaCN_2_ further enhanced plant vigour, bulb development, and nitrogen use efficiency [21]. Additionally, CaCN_2_ improved bulb nutritional quality by increasing phenolic and flavonoid concentrations, antioxidant activity, and essential mineral content [22]. However, excessive application rates of CaCN_2_ (e.g., 600 kg ha^−1^) were associated with postharvest issues such as sprouting and rot, highlighting the importance of optimized dosing strategies [22].

From an environmental standpoint, CaCN_2_ offers distinct advantages. Under simulated rainfall conditions, nitrogen leaching losses from CaCN_2_ (130 kg ha^−1^ N) were as low as 4.2% in clay soils, substantially lower than those observed with urea or LAN, particularly on steep slopes prone to runoff [22]. Moreover, its biocidal properties reduce the need for chemical herbicides and fungicides, supporting more integrated and sustainable crop management systems [9,23]. These findings collectively position CaCN_2_ as a viable and environmentally responsible alternative to conventional nitrogen fertilizers in both leafy and bulb vegetable production systems.

Despite these promising attributes, a comprehensive synthesis of CaCN_2_’s agronomic and environmental performance remains limited. Most studies have focused on isolated aspects such as nitrogen efficiency, disease suppression, or soil pH modification. A unified, critical review that integrates these findings and evaluates CaCN_2_’s comparative performance across diverse agroecological contexts is lacking. Moreover, regional variability in soil types, climate, and cropping systems limits the generalizability of existing data, highlighting the need for context-specific research and recommendations.

This review aims to bridge that gap by providing a comprehensive evaluation of calcium cyanamide as an alternative nitrogen fertilizer. It synthesises current knowledge on its agronomic benefits, environmental behaviour, and multifunctional roles in sustainable cropping systems. As agriculture faces increasing pressure to reduce its ecological footprint while maintaining productivity, CaCN_2_ emerges as a promising tool for climate-smart and regenerative farming.

By critically analysing the available evidence and identifying key areas for further research, this review seeks to inform researchers, extension agents, and practitioners about the potential of CaCN_2_ to enhance nitrogen use efficiency, reduce environmental harm, and support resilient vegetable production systems. Its reintroduction into modern agriculture represents a significant step toward more integrated, efficient, and ecologically responsible fertilization strategies.

## 2. Chemical Properties and Soil Transformations

### 2.1. Composition and Physicochemical Characteristics

Calcium cyanamide (CaCN_2_), commonly supplied in granular, dust-reduced formulations (e.g., Perlka^®^), typically contains ~19.8% total N and >50% CaO, conferring both nitrogen supply and a net liming capacity per unit product and per unit N applied [24,25,26,27]. Modern commercial granules are wax-coated to reduce fines, moisture uptake, and premature hydrolysis during handling and storage, improving application uniformity relative to legacy powdered forms [26,27,28].

From a physicochemical standpoint, CaCN_2_ is sparingly soluble in water but reactive in moist soils, where hydrolysis proceeds rapidly once the fertilizer is incorporated and wetted. The accompanying release of Ca(OH)_2_ contributes to a local pH rise at the reaction front, influencing subsequent chemical and enzymatic steps [25,29,30,31,32]. Typical industrial specifications also include minor fractions of nitrate-N and dicyandiamide (DCD) present at manufacture, alongside DCD formed in situ during transformation [24,28].

### 2.2. Sequential Transformation Pathway in Moist Soil

Following incorporation and wetting, CaCN_2_ undergoes a multi-step transformation that regulates nitrogen speciation in the soil solution and exchange complex:

(i) Initial hydrolysis to hydrogen cyanamide and calcium dihydroxide

CaCN_2_ + 2H_2_O → Ca(OH)_2_ + H_2_CN_2_

The product H_2_CN_2_ (hydrogen cyanamide) is a reactive intermediate; co-formation of Ca(OH)_2_ drives a local increase in pH, characteristic of CaCN_2_’s liming action [25,26,32].

(ii) Conversion of cyanamide to urea and dicyandiamide (DCD)

H_2_CN_2_ → CO(NH_2_)_2_ + DCD

This step closes the highly reactive cyanamide phase and introduces DCD, a recognised nitrification inhibitor that slows the microbial oxidation of NH_4_^+^ to NO_3_^−^ [25,28].

(iii) Urea hydrolysis to ammonium (enzyme-mediated)

CO(NH_2_)_2_ + H_2_O → 2NH_4_^+^ + CO_2_

Generated NH_4_^+^ is less mobile than nitrate and is subject to exchange adsorption; the local pH (from Ca (OH)_2_) can further influence speciation and sorption [31,33,34,35].

(iv) Nitrification: ammonium → nitrate

NH_4_^+^ → NO_3_^−^ (Nitrosomonas/Nitrobacter)

This oxidation is delayed in CaCN_2_-amended soils by DCD, which inhibits the ammonia-oxidising step; the liming environment also modulates microbial activity and enzyme kinetics [25,28,36,37,38].

Time-course observations reported for the cyanamide → urea step range ~7–14 days under typical field moisture/temperature conditions; urea hydrolysis follows rapidly in biologically active soils, while nitrification is slowed proportionally to DCD presence, temperature, and moisture regime [25,26,32,33,36].

### 2.3. Kinetics and Controlling Soil Factors (pH, Moisture, Temperature, Matrix)

The rates and relative dominance of the reactions outlined in Section 2.2 are highly condition-dependent:

- Moisture: Hydrolysis of Calcium cyanamide (CaCN_2_) and subsequent cyanamide transformations require adequate soil water. Dry or intermittently wetted soils prolong the cyanamide phase and slow progression to urea/NH_4_^+^, extending the time window in which reactive intermediates are present [25,32,33].
- pH and liming front: Co-formation of Ca(OH)_2_ elevates local pH, which can (i) affect cyanamide condensation/polymerisation equilibria, (ii) alter urease activity, and (iii) shift sorption–desorption behaviour of NH_4_^+^/NH_3_. The liming front propagates with diffusion and mixing, creating micro-zones where kinetics differ from bulk soil [25,31,33,38].
- Temperature: Reaction and enzyme rates increase with temperature; in cool soils, cyanamide persistence and the interval before safe availability of NH_4_^+^/NO_3_^−^ are extended, whereas warm, moist conditions compress the sequence toward urea → NH_4_^+^ formation [25,32,33].
- Soil texture and organic matter: In coarse-textured (sandy) matrices, faster infiltration may speed hydrolysis but reduce residence time of intermediates; fine-textured or high-OM soils provide greater adsorption capacity (particularly for NH_4_^+^), influencing temporary partitioning of N forms and the spatial distribution of the liming effect [24,31,33].

Collectively, these factors explain field variability in the observed timing between application and the appearance of plant-available NH_4_^+^/NO_3_^−^, and they underpin practical guidance (timing and incorporation) provided later in the manuscript to prevent phytotoxic exposure to cyanamide [25,26,32,33,38].

### 2.4. Intermediates and By-Products: Hydrogen Cyanamide and Dicyandiamide (DCD)

Two intermediates are central to CaCN_2_ chemistry in soil:

- Hydrogen cyanamide (H_2_CN_2_): The immediate hydrolysis product; reactive and short-lived under favourable moisture/temperature. Its chemical reactivity underpins transient biocidal behaviour noted historically, but in Chapter Two, we emphasise chemistry: rapid conversion reduces persistence when moisture and temperature are adequate [25,26,32].
- Dicyandiamide (DCD): Present as a minor manufactured fraction and formed in situ during cyanamide transformation. DCD inhibits ammonia oxidation (first step of nitrification), thereby delaying NO_3_^−^ formation and prolonging the NH_4_^+^ phase. The extent and duration of inhibition depend on DCD concentration, temperature, and microbial community responsiveness [28].

These intermediates define the temporal speciation of N and the lag to nitrate formation following CaCN_2_ application. Their appearance/disappearance is constrained by the same environmental drivers outlined in Section 2.3 [25,28,32].

### 2.5. Nitrogen Speciation and Partitioning (Chemical Perspective)

Within the transformation sequence, soil nitrogen cycles through cyanamide → urea → ammonium → nitrate. From a chemical vantage:

- Urea → NH_4_^+^ is enzyme-catalysed and typically rapid in biologically active soils; NH_4_^+^ can adsorb to exchange sites, reducing immediate mobility and shifting equilibria with NH_3_ depending on pH [31,33].
- NH_4_^+^ → NO_3_^−^ is microbially mediated; the presence of DCD introduces an induction period before nitrate predominates. The local liming effect can additionally modulate enzyme activity and sorption, indirectly influencing the NH_4_^+^/NO_3_^−^ balance [25,28,38].
- The net liming provided by Ca(OH)_2_ may alter carbonate equilibria and buffering capacity over the short term, though bulk pH changes depend on dose, soil buffering, and mixing [24,25,31].

This speciation pathway is foundational for later sections (Agronomic Benefits and Environmental Impacts), where plant uptake, leaching potential, and emission pathways are analysed; here we confine the discussion to chemical transformations and partitioning [25,31,33].

### 2.6. Handling Chemistry and Stability

Because Calcium cyanamide (CaCN_2_) is reactive in the presence of water, storage and application protocols seek to minimise premature hydrolysis and worker exposure to reactive intermediates. Granular, wax-coated products show improved stability, reduced dust, and unlimited shelf life if kept dry and sealed; moisture ingress can initiate hydrolysis and release alkaline reaction products and volatile species linked to subsequent steps (e.g., NH_3_ from NH_4_^+^/NH_3_ equilibria in high-pH microsites) [39,40]. These aspects are operational rather than agronomic; they are summarised here to complete the chemical profile of the product and are referenced more fully in later practical guidelines [39,40,41].

## 3. Agronomic Benefits

Calcium cyanamide (CaCN_2_) is recognised for its unique nitrogen-release profile, which aligns well with the physiological demands of crops. Upon application to moist soil, CaCN_2_ undergoes a multi-step transformation through hydrogen cyanamide, urea, ammonium (NH_4_^+^), and finally nitrate (NO_3_^−^) [20,26,38]. This gradual conversion ensures a sustained nitrogen supply and reduces nutrient losses via leaching or volatilization. The presence of dicyandiamide (DCD), a nitrification inhibitor formed during CaCN_2_ breakdown, further delays the conversion of NH_4_^+^ to NO_3_^−^ and enhances nitrogen use efficiency [26,38]. This slow-release behaviour is particularly advantageous in high-rainfall or irrigated systems where conventional nitrogen fertilizers are prone to rapid leaching.

### Agronomic Performance: Slow-Release Effects, Yield, and Quality

Calcium cyanamide’s slow-release nitrogen behaviour differs from highly soluble fertilizers such as urea, limestone ammonium nitrate (LAN), and calcium ammonium nitrate (CAN). Comparative studies show that CaCN_2_ releases nitrogen gradually through the cyanamide → urea → NH_4_^+^ → NO_3_^−^ sequence, increasing NH_4_^+^ residence time and lowering losses relative to urea or ammonium-nitrate programmes. Pleysier and de Willigen [42] reported higher nitrogen efficiency of CaCN_2_ across multiple soils versus urea/ammonium nitrate due to delayed nitrification and improved retention. Suzuki et al. [38] demonstrated that CaCN_2_ increased soil pH, reduced nitrification gene abundance, and lowered N_2_O emissions compared with urea. Consistent with these observations, meta-evidence on nitrification inhibitors indicates that DCD (formed in situ during CaCN_2_ transformation) slows the NH_4_^+^ → NO_3_^−^ conversion, reducing leaching and improving nitrogen use efficiency [13]. Collectively, these studies indicate that CaCN_2_ provides more stable nitrogen availability than urea, LAN, or CAN, especially in leaching-prone environments.

Numerous field studies show that CaCN_2_ improves nitrogen use efficiency while enhancing crop yield and quality. In leafy vegetables such as lettuce, CaCN_2_ reduces nitrate accumulation in edible tissues while maintaining or increasing biomass [19]. In curly endive, pre-transplant CaCN_2_ at 100 kg ha^−1^ N significantly increased fresh head weight, visual quality, ascorbic acid, and total phenolics, with the strongest effects during spring–summer cycles when nitrogen losses are higher [18]. Strawberry grown on CaCN_2_-treated soil exhibited greater vegetative growth, higher antioxidant capacity, and improved fruit quality [20]. Grafted melon and eggplant systems also showed enhanced vigour and yield, with reduced symptoms of *Fusarium* and *Verticillium* wilts, following CaCN_2_ application [23].

Across diverse soils and environments, CaCN_2_ provides multifaceted agronomic benefits. In onion production, CaCN_2_ improves bulb size, uniformity, and marketable yield via synchronised nitrogen supply [21]. In Australian calabrese (broccoli) trials, banded CaCN_2_ reduced clubroot (*Plasmodiophora brassicae*) and doubled marketable yields relative to untreated controls, illustrating phytosanitary value alongside nutrition [39]. These advantages are pronounced in sandy-loam soils, which are susceptible to leaching and have low buffering capacity. CaCN_2_ has been shown to increase soil pH, shift microbial communities, reduce nitrification/denitrification gene abundance, and lower N_2_O emissions [38]. During its transient cyanamide phase, CaCN_2_ also exhibits fungicidal, herbicidal, and molluscicidal activity against pathogens such as *Sclerotinia sclerotiorum*, *Phytophthora capsici*, and *P. brassicae* [40]. In composting systems, CaCN_2_ enhances sanitation and total nitrogen retention—effects that may translate to low-CEC field soils [41].

On sandy-loam soils in South Africa, CaCN_2_ significantly increased total and marketable onion yields, improved bulb uniformity, and enhanced nitrogen use efficiency relative to urea and ammonium-nitrate programmes [21]. CaCN_2_-treated onions also displayed improved storability, firmer bulbs, and reduced postharvest decay [22]. In addition, CaCN_2_ contributes to soil disinfection, pH buffering, and improved microbial balance, supporting nutrient cycling and recovery of soil function in coarse-textured soils [42,43]. Overall, agronomic benefits extend beyond yield to include improved uniformity, reduced physiological disorders, and enhanced postharvest quality, arising from the combined effects of slow-release nitrogen, calcium supply, transient sanitation, and liming-driven changes to soil structure and microbial activity [26,43].

## 4. Environmental Impacts

### 4.1. Nitrogen Use Efficiency (NUE)

Calcium cyanamide (CaCN_2_) improves nitrogen use efficiency (NUE) through both recovery and physiological pathways. Its slow-release transformation sequence—cyanamide → urea → ammonium (NH_4_^+^) → nitrate (NO_3_^−^)—provides nitrogen in closer synchrony with crop demand, thereby reducing losses and increasing plant uptake compared with more soluble nitrogen sources [25,28,44]. During this process, dicyandiamide (DCD) forms in situ and acts as a nitrification inhibitor, delaying the conversion of NH_4_^+^ to NO_3_^−^ and lowering losses via leaching and denitrification [44,45,46].

Physiologically, CaCN_2_ programs are often associated with enhanced nitrogen assimilation and lower nitrate accumulation in edible tissues [47]. For example, lettuce and curly endive fertilized with CaCN_2_ have consistently shown reduced tissue nitrate compared with ammonium nitrate or urea, contributing to improved food safety and quality [18,19]. In addition, short-day onion trials report higher marketable yield and improved nitrogen-use metrics when CaCN_2_ is used as a pre-plant source, particularly on leaching-prone soils [21,22]. The localized liming micro-environment generated by Ca(OH)_2_ during CaCN_2_ hydrolysis can further modulate enzyme activity and microbial pathways in acidic or coarse-textured soils, supporting higher NUE where leaching pressure is elevated [25,28,44]. To maintain these benefits and limit NH_3_ volatilization on high-pH surfaces, CaCN_2_ should be incorporated into moist soil and followed by light irrigation where appropriate [24,25,31,33].

### 4.2. Leaching and Emissions

CaCN_2_’s most compelling environmental advantage is its ability to reduce nitrate leaching, particularly under high rainfall or intensive irrigation [21]. Long-term studies in Portugal and tropical environments found that CaCN_2_-treated soils retained nitrogen more effectively than soils fertilized with ammonium nitrate or urea; in some trials, nitrate leaching losses were reported as low as ~3% of applied N with CaCN_2_—substantially lower than conventional programs [48,49]. Under simulated rainfall and in field comparisons, clay soils receiving CaCN_2_ also exhibited low loss fractions when dosing and timing were optimized [22].

Calcium cyanamide can additionally lower nitrous oxide (N_2_O) emissions. In Japanese vegetable systems, lime-nitrogen applications significantly reduced N_2_O fluxes, with pronounced effects in poorly drained soils [30]. These reductions arise from the DCD-mediated delay of nitrification, which limits nitrate availability for denitrifiers, and from the liming effect that alters microbial pathway kinetics [33,38].

Examples of crop systems benefiting from CaCN_2_ in leaching/emissions contexts include curly endive [18], organic tomato with weed/disease control co-benefits [19], strawberry in protected cultivation [20], short-day onion under rainfall/irrigation [21,22], and calabrese/broccoli in clubroot-affected soils where CaCN_2_ improves sanitary status and marketable yield [39]. Because temperature and moisture influence both transformation rates and inhibitor persistence, pre-plant intervals should be adjusted to local conditions to sustain leaching and emissions reductions [25,33,38]. Benefits are generally larger in sandy or coarse-textured soils (high leaching risk) and poorly drained fields (higher denitrification potential) [21,48,49].

### 4.3. Biocidal and Disease Suppression Effects

Beyond nutrient management, CaCN_2_ exhibits biocidal properties that can reduce reliance on pesticides. In moist soil, the transient hydrogen-cyanamide phase creates a short-lived phytotoxic environment that inhibits pathogen sporulation and weed seed germination [27,42]. Suppression has been documented for *Plasmodiophora brassicae* (clubroot), *Sclerotinia sclerotiorum* (white rot), *Fusarium oxysporum*, *Verticillium* spp., and *Olpidium bornovanus* [50,51,52,53]. These outcomes are mediated by both direct toxicity during the cyanamide phase and indirect stimulation of beneficial or antagonistic microbial groups [54]. CaCN_2_ use has also been associated with greater bacterial diversity and higher extracellular enzyme activities (e.g., amylase, protease, phosphatase), features commonly linked to soil suppressiveness [26,37].

Examples of crops reporting phytosanitary or quality co-benefits include brassicas (clubroot suppression; higher marketable yield) [39], strawberry (higher fruit quality and antioxidant traits) [20], onion (improved bulb uniformity and storability) [22], and organic tomato (integrated weed and soil-borne disease management alongside fertilization) [19]. CaCN_2_ also provides weed suppression—especially in pre-plant applications—by inhibiting seed germination, and it has been incorporated into integrated weed-management programs to reduce herbicide inputs [40,55,56]. To translate these benefits safely into practice, observe pre-plant waiting intervals so the cyanamide phase dissipates before seeding or transplanting; integrate CaCN_2_ with rotation, straw/organic amendments, or solarization where suitable to support microbial recovery and long-term soil function [20,37,42]. Timing and dose should align with crop sensitivity and local soil moisture/temperature to avoid phytotoxicity while maximizing sanitation and environmental gains [25,42].

## 5. Soil Health and Microbial Balance

Soil health is a cornerstone of sustainable agriculture, encompassing physical structure, chemical fertility, and biological activity [22]. The soil microbial community plays a pivotal role in nutrient cycling, organic matter decomposition and disease suppression. However, intensive agricultural practices, particularly the overuse of soluble synthetic nitrogen fertilizers can disrupt microbial diversity and function, reducing soil resilience and productivity [36,37,56].

### 5.1. Calcium Cyanamide and Soil Microbial Dynamics

Calcium cyanamide (CaCN_2_) is a multifunctional input that supplies nitrogen while modulating soil microbial ecology. After application, CaCN_2_ hydrolyzes to cyanamide and subsequently to urea, ammonium (NH_4_^+^) and nitrate (NO_3_^−^); during these transformations, dicyandiamide (DCD) forms in situ and acts as a nitrification inhibitor. By delaying NH_4_^+^ → NO_3_^−^, CaCN_2_ reduces nitrogen losses and alters microbial nitrogen-cycling pathways [21,38].

Microcosm and field observations indicate that CaCN_2_ can suppress nitrous oxide (N_2_O) emissions and shift bacterial community composition relative to urea-based programs. A local pH rise (via Ca(OH)_2_) is often associated with reduced activity or abundance of nitrifiers and denitrifiers, consistent with lower N_2_O potential and altered nitrogen transformations [23,38]. Studies have also reported increases in extracellular enzyme activities (e.g., amylase, protease, phosphatase) and changes in microbial network connectivity following CaCN_2_ use, suggesting enhanced nutrient turnover once the transient biocidal phase subsides [26,37].

Examples of crop systems showing microbial or functional improvements under CaCN_2_ include brassicas with clubroot pressure (enhanced suppressiveness and marketable yield) [39], organic tomato, where weed and soil-borne disease management co-benefits accompany fertilization [19], and strawberry in protected cultivation with improved plant vigor and antioxidant capacity [20].

### 5.2. Microbial Recovery and Soil Fertility

Although the cyanamide phase is transiently biocidal, effects are dose- and time-dependent rather than uniformly detrimental. In cucumber systems, CaCN_2_ combined with pepper straw improved yield and soil fertility while allowing microbial communities to recover during the growing season [39]. Populations of culturable bacteria and actinomycetes typically rebound within weeks, reflecting microbial resilience and restoration of soil biological activity [37]. Integrating CaCN_2_ with organic amendments (e.g., straw return, composts) and observing pre-plant waiting intervals (see Section 6) helps balance sanitation benefits with rapid reestablishment of beneficial microbiota [15,36]. Examples highlighting recovery and fertility gains include cucumber with pepper straw (yield and fertility improvements alongside microbial recovery) [39], strawberry systems where microbial diversity and nutrient turnover increase during the season [20], and onion rotations that pair CaCN_2_ with residue management to support microbial rebound [22].

### 5.3. Disease of Suppression and Microbial Balance

Beyond nutrient dynamics, CaCN_2_ has demonstrated efficacy against soil-borne pathogens such as *Fusarium oxysporum* and *Plasmodiophora brassicae*. Shi et al. [36] reported lower pathogen abundance alongside increases in beneficial microbial groups, indicating a shift toward suppressive soils. In strawberry systems, CaCN_2_ treatment reduced seedling mortality by lowering pathogen loads and increasing beneficial taxa (e.g., actinomycetes, Alphaproteobacteria), while enhancing bacterial network connectivity and soil nutrient availability [37].

Illustrative crop examples include brassicas (clubroot suppression; higher marketable yield) [39], strawberry (lower seedling mortality and improved quality traits) [20,36], and onion (improved bulb uniformity and storability when phytosanitary effects complement nutrient management) [22].

Align CaCN_2_ dose and timing with crop sensitivity, maintain pre-plant waiting intervals, and pair applications with organic amendments or residue returns. This approach sustains the sanitation benefits of the cyanamide phase while supporting rapid microbial recovery and long-term soil function [15,36].

## 6. Practical Considerations and Limitations

Calcium cyanamide (CaCN_2_) delivers agronomic and environmental benefits, but disciplined implementation is essential to avoid phytotoxicity and to protect soil biological function. This section consolidates the key practices when to apply, how to incorporate, and how to integrate CaCN_2_ within broader programs so that efficacy is maximized while risks are minimized.

### 6.1. Application Timing and Incorporation Requirements

Effective use of CaCN_2_ begins with timing. In moist soil, CaCN_2_ hydrolyses to hydrogen cyanamide a short-lived, biocidal intermediate before transforming to urea and then plant-available forms. To protect emerging seedlings, CaCN_2_ should be incorporated into the soil, followed by irrigation, and allowed sufficient time to transform before planting [57,58]. Incorporation ensures uniform distribution and reduces volatilization losses; surface-only applications, especially on alkaline soils, increase the risk of NH_3_ loss and localized injury [24,25,31,33].

As a practical rule, apply CaCN_2_ at least 14 days before sowing or transplanting, with longer intervals in cool or dry conditions. In transplanted crops, split programs are common for example, ~500 kg ha^−1^ as a base dressing, followed by a second application 14–21 days after transplanting—provided the first application is safely transformed by transplant time [59]. To further manage risk, band placement can keep the cyanamide reaction zone away from the seed line in sensitive crops, while deeper incorporation is appropriate for robust crops and heavier soils [24,25,31,33]. Specific guidelines for different crops are presented in Table 1.

Integration into whole-farm practice also matters. CaCN_2_ can be paired with rotations and organic amendments (e.g., straw or compost) or solarization to enhance short-term sanitation while supporting microbial recovery. In high-rainfall systems, coordinate CaCN_2_ with nitrification management and irrigation scheduling to sustain nitrogen use efficiency and curb losses. Always separate CaCN_2_ from strong acids and from sensitive seedlings in both time (a clear pre-plant interval) and space (banding or deeper placement). Align the application with transplant dates and anticipated weather so the cyanamide phase has fully dissipated by the time plants are established [20,37,42,57].

### 6.2. Waiting Periods to Avoid Phytotoxicity and Crop Sensitivity

The phytotoxicity of hydrogen cyanamide necessitates a waiting interval between CaCN_2_ application and planting to allow conversion into less toxic forms such as urea and ammonium. Failure to observe this interval can result in poor germination, stunted growth, and root damage, particularly in sensitive crops [42,55]. The required interval depends on soil moisture, temperature, and microbial activity; degradation is slower under cool or dry conditions. Sensitive crops—especially at early stages—include lettuce, spinach, and young brassica seedlings, which may exhibit chlorosis, root inhibition, and reduced vigour if exposed too soon after application [25,59]. Risk mitigation includes applying during fallow or preceding with tolerant species or using CaCN_2_ as a pre-plant soil-conditioning step to suppress pathogens and weeds once the cyanamide phase has dissipated [20,37,42].

Crop-specific waiting-interval notes. Leafy greens: strict pre-plant interval; incorporate and irrigate. Brassicas: observe full interval; favour band placement. Onions: respect intervals to avoid early stress. Strawberry transplants: apply pre-plant with sufficient lead time for dissipation [21,22,25].

### 6.3. Storage and Handling Precautions

Commercial CaCN_2_ is supplied as a granular, dust-reduced (often wax-coated) product to enhance stability and reduce handling risks; when stored dry in sealed packaging it has an unlimited shelf life [60]. Because CaCN_2_ is reactive with moisture, key precautions include avoid inhalation and skin contact (use gloves, eye protection, and a dust mask or respirator); store dry in a well-ventilated area with bags sealed and off the floor; and do not mix with acidic fertilizers or materials, which can accelerate decomposition and increase phytotoxicity [60,61]. If the product becomes damp, handle with caution and avoid application near sensitive crops or immediately prior to seeding or transplanting [60,61].

## 7. Comparative Analysis with Conventional Fertilizers

### 7.1. Agronomic Performance vs. Urea, LAN, and CAN

Calcium cyanamide (CaCN_2_) has been widely evaluated against conventional nitrogen fertilizers such as urea, limestone ammonium nitrate (LAN), and calcium ammonium nitrate (CAN) in terms of yield, nitrogen use efficiency (NUE), and disease suppression. In vegetable crops such as lettuce, onion, and brassicas, CaCN_2_ performs comparably to or better than urea and CAN [18,42]. In calabrese (broccoli), CaCN_2_ application resulted in a doubling of marketable yield compared with untreated controls and outperformed urea in disease-susceptible soils [39]. CaCN_2_ also enhances soil health by increasing microbial diversity and suppressing soil-borne pathogens such as *Plasmodiophora brassicae* and *Fusarium oxysporum*, benefits not typically associated with urea or LAN [36,37].

Mechanistically, CaCN_2_’s staged transformation from cyanamide to urea, ammonium, and nitrate results in sustained ammonium availability and delayed nitrification due to dicyandiamide (DCD) formed in situ. This reduces leaching risk and supports higher plant uptake relative to soluble nitrogen fertilizers [13,38]. These advantages are most evident under leaching-prone or disease-susceptible conditions, such as coarse-textured soils or high-rainfall systems [18,21,22,39].

### 7.2. Economic and Environmental Trade-Offs

Economically, CaCN_2_ is generally more expensive per unit nitrogen than urea or LAN due to its manufacturing process and smaller market scale [60]. However, its multifunctional roles as a nitrogen source, liming material, and phytosanitary agent can reduce the need for additional lime, fungicides, and herbicides, partially offsetting its higher cost [25].

Environmentally, CaCN_2_ provides significant advantages. It decreases nitrate leaching and nitrous oxide (N_2_O) emissions because of the natural formation of DCD during its transformation in soil [30,45]. In long-term studies conducted in Portugal and tropical regions, CaCN_2_-treated soils retained over 90% of applied nitrogen, whereas urea and CAN programs showed substantially higher losses [49,60]. These benefits make CaCN_2_ a strong candidate for systems facing nutrient losses due to rainfall, irrigation, or poor soil structure [21,22,30,38].

## 8. Research Gaps and Future Directions

Despite its agronomic and environmental benefits, several research gaps remain before CaCN_2_ can be widely adopted in modern, sustainable agricultural systems.

- Long-term, multi-location trials. Current studies often focus on specific crops or localized systems. Broader trials across diverse agroecological zones are needed to evaluate CaCN_2_ performance under varying soil, climate, and management conditions [20,42].
- Integration with organic and regenerative systems. CaCN_2_’s disease-suppressive and soil-conditioning properties offer potential for regenerative agriculture, but its compatibility with organic certification remains uncertain. Research on blending CaCN_2_ with composts, cover crops, and microbial inoculants is needed [36,37].
- Climate-smart agriculture (CSA) potential. Given CaCN_2_’s capacity to reduce nitrate leaching and N_2_O emissions, its role in CSA strategies such as greenhouse-gas mitigation and soil-carbon dynamics should be evaluated in long-term field studies [30,60].
- Decision-support tools. Effective use of CaCN_2_ requires precise timing. Decision tools integrating weather forecasts, soil data, and crop phenology could improve recommendations for waiting intervals, incorporation depth, and split applications.
- Crop-specific sensitivity thresholds. Further work is required to define safe timing, dose, and band placement for sensitive crops such as leafy vegetables, young brassicas, and strawberries [21,25].
- Transformation kinetics under field variability. More research is needed on cyanamide → urea → NH_4_^+^ → NO_3_^−^ conversion under different pH, temperature, and moisture regimes to refine pre-plant interval guidance [25,33].
- Comparative meta-analysis. Synthesizing results across systems to compare CaCN_2_ with urea, LAN, and CAN for yield, quality, NUE, nitrate leaching, and N_2_O/NH_3_ emissions would help define benefit ranges and strengthen agronomic recommendations [13,38,49,60].

## Figures and Tables

**Table 1:** Crop-specific timing and incorporation guidelines for CaCN_2_ (indicative best practices).

Crop/System	When to Apply	Incorporation Depth/Placement	Minimum Waiting Interval *	Notes
Leafy greens (lettuce, spinach)	Pre-plant, before bed formation	Shallow incorporation; band away from seed line	≥14 days	Strict interval to avoid seedling stress; irrigate after incorporation [18,19,24,25,31,33].
Brassicas/broccoli	Pre-plant	Banded placement 5–10 cm from seed line	≥14 days	Banding reduces early contact; clubroot contexts may benefit from sanitation co-effects [39].
Onions (direct-seeded or transplants)	Pre-plant; optional split with early topdress	Incorporate into bed zone; avoid seed line	≥14 days	Improves bulb uniformity; light irrigation after incorporation; optional split after establishment [21,22].
Strawberry (transplants)	Pre-plant, bed preparation	Incorporate across bed profile	≥14 days	Allow full dissipation before transplanting; quality and vigour benefits reported [20].
Organic/processing tomato (transplants)	Pre-plant	Uniform incorporation; avoid transplant holes	≥14 days	Supports weed and soil-borne disease suppression; confirm dissipation before transplanting [19,42].

* Extend the waiting interval under cool or dry conditions; shorten only where warm, moist, and biologically active soils accelerate transformation [25,31].

## Data Availability

Data supporting this work will be made available upon request.
